# RNA-Seq reveals changes in human placental metabolism, transport and endocrinology across the first–second trimester transition

**DOI:** 10.1242/bio.058222

**Published:** 2021-06-08

**Authors:** Malwina Prater, Russell S. Hamilton, Hong Wa Yung, Andrew M. Sharkey, Paul Robson, N. Erlyani Abd Hamid, Eric Jauniaux, D. Stephen Charnock-Jones, Graham J. Burton, Tereza Cindrova-Davies

**Affiliations:** 1 Centre for Trophoblast Research, Department of Physiology, Development and Neuroscience, University of Cambridge, Downing Street, Cambridge, CB2 3EG, UK; 2 Department of Genetics, University of Cambridge, Downing Street, Cambridge, CB2 3EH, UK; 3 Department of Pathology, University of Cambridge, Tennis Court Road, Cambridge, CB2 1QP, UK; 4 The Jackson Laboratory, The JAX Center for Genetics of Fertility and Reproduction, 10 Discovery Drive, Farmington, CT 06032, USA; 5 Genome Institute of Singapore, Singapore 138672, Singapore; 6 Department of Obstetrics and Gynaecology, EGA Institute for Women's Health, Faculty of Population Health Sciences, University College London, London, WC1E 6BT, UK; 7 Department of Obstetrics and Gynaecology, University of Cambridge, The Rosie Hospital, Cambridge, CB2 0SW, UK; 8 National Institute for Health Research, Cambridge Biomedical Research Centre, Cambridge CB2 0QQ, UK

**Keywords:** Early placenta, Metabolism, Transcriptome, Human pregnancy, Methylation

## Abstract

The human placenta is exposed to major environmental changes towards the end of the first trimester associated with full onset of the maternal arterial placental circulation. Changes include a switch from histotrophic to hemotrophic nutrition, and a threefold rise in the intraplacental oxygen concentration. We evaluated their impact on trophoblast development and function using RNA-sequencing (RNA-Seq) and DNA-methylation analyses performed on the same chorionic villous samples at 7–8 (*n*=8) and 13–14 (*n*=6) weeks of gestation. Reads were adjusted for fetal sex. Most DEGs were associated with protein processing in the endoplasmic reticulum (ER), hormone secretion, transport, extracellular matrix, vasculogenesis, and reactive oxygen species metabolism. Transcripts higher in the first trimester were associated with synthesis and ER processing of peptide hormones, and glycolytic pathways. Transcripts encoding proteins mediating transport of oxygen, lipids, protein, glucose, and ions were significantly increased in the second trimester. The motifs of CBX3 and BCL6 were significantly overrepresented, indicating the involvement of these transcription factor networks in the regulation of trophoblast migration, proliferation and fusion. These findings are consistent with a high level of cell proliferation and hormone secretion by the early placenta to secure implantation in a physiological low-oxygen environment.

## Abstract (precis)

**Summary:** Full onset of the maternal circulation to the human placenta at 10–12 weeks of gestation is associated with transcript changes indicative of switches in protein processing, hormone secretion, transport and vasculogenesis.

## INTRODUCTION

The placenta is essential to a successful pregnancy and the lifelong health of the offspring. Impaired placental function has both immediate obstetric consequences (Brosens et al., 2011), including miscarriage, fetal growth restriction, pre-eclampsia and stillbirth, and long-term impact on the risk of chronic disease for the offspring (Burton et al., 2016). Recent advances in imaging and biomarker studies indicate that the pathophysiology of many non-communicable complications of pregnancy starts during early pregnancy (Smith, 2010). The human gestational sac from which the placenta and fetus develop undergoes a major transition towards the end of the first trimester with the switch from histotrophic to hemotrophic nutrition (Burton et al., 2010). This transition, which involves the same placental structure being supported by contrasting nutrient pathways, is unique to humans and great apes, and may explain why conditions such as pre-eclampsia are virtually restricted to the human.

During the first trimester, maternal arterial blood flow into the placenta is restricted by aggregates of endovascular trophoblast that migrate down the lumens of the endometrial spiral arteries (Turco and Moffett, 2019). Consequently, the placental tissues develop in a relatively low-oxygen environment (Jauniaux et al., 2000), supported principally by carbohydrate- and lipid-rich secretions from the endometrial glands (Burton et al., 2002). These secretions are also a potential source of mitogenic growth factors, including epidermal growth factor that stimulates proliferation of cytotrophoblast cells when applied to explant cultures (Burton et al., 2020; Maruo et al., 1992). Metabolism of the placental tissues is heavily glycolytic, supported by high activity in polyol metabolic pathways (Burton et al., 2017).

Towards the end of the first trimester, the endovascular aggregates canalise, which, along with coordinated remodelling of the arcuate and radial arteries (Allerkamp et al., 2020), allows full onset of the maternal circulation as confirmed by a threefold rise in the intraplacental oxygen concentration (Jauniaux et al., 2000). The rise is thought to induce maturational changes, including villous regression to form the definitive placenta and the membranes (Jauniaux et al., 2003b). Oxygen has also been implicated in the regulation of trophoblast proliferation and invasion, hormone production and transporter expression, based largely on *in vitro* data (Caniggia et al., 2000; Graham et al., 2000; Pringle et al., 2010). The transition from histotrophic to hemotrophic nutrition involves other potential influences, however, such as the dilution of growth factor support and increased biomechanical forces, including shear stress, at the villous surface. Previous studies have compared gene expression in the first trimester placenta with that of term placentas using microarray analysis (Mikheev et al., 2008; Sitras et al., 2012). In order to address the critical changes taking place during the first–second trimester transition, we performed RNA-Seq and array-based DNA methylation profiling on the same samples of placental villous tissue, obtained from accurately dated and narrow windows of gestation: 7–8 weeks and 13–14 weeks. The samples were obtained under optimal conditions using an ultrasound-guided chorionic villous sampling (CVS) technique that avoided the stress induced by curettage (Cindrova-Davies et al., 2015). We focussed our analyses on transcripts encoding proteins involved in metabolism, hormone synthesis, transport and cell proliferation. We also discuss transcripts with both differential expression and differentially methylated regions found in their promoters and gene-bodies.

## RESULTS

**Fig. 1.**
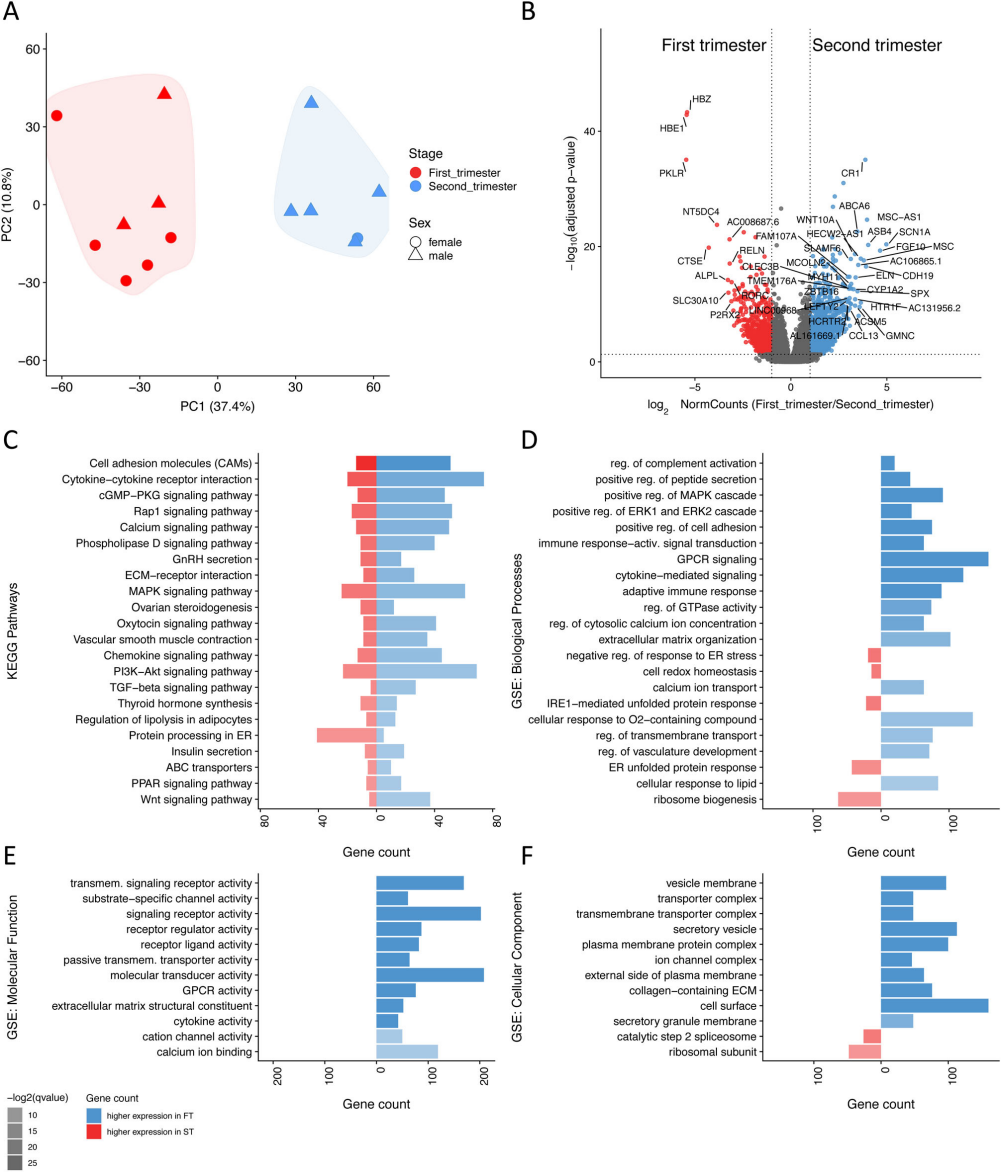
**RNA-Seq identification of DEGs, functions and pathways distinct to first and second trimester placentas.** (A) PCA separation of first and second trimester samples. (B) Volcano plot of DEGs, with genes higher in the first trimester in red, and those higher in the second trimester in blue. (C) Selected Kegg pathways, which were differentially regulated during the first and second trimester. (D–F) Barplots showing selected differentially regulated GO terms related to biological processes (D), molecular functions (E) and cellular components (F). Each barplot shows how many genes within each term are expressed more in the first (red) or second trimester (blue). Transparency is used to show the most significant (*P*adj.) terms as least transparent. Terms were ordered by q-value.

Samples separated clearly on the basis of gestational age with both principal component analysis (PCA) and hierarchical clustering (Fig. 1A; Fig. S1). Differential expression analysis identified 3260 differentially expressed genes (DEG) (adjusted *P*-value, *P*adj. ≤0.05, absolute fold change ≥2; Fig. 1B; Table S1). In view of the sex bias in the sample groups (Fig. 1A), we performed a sex-adjustment analysis. Sex was confirmed using sex-specific genes, *Xist, Rps4y1, Ddx3y, Usp9y* and *Sry,* and was included in the design formula (∼sex+condition) as a blocking factor to account for variation in the data.

Using Kegg pathway analysis and Gene Ontology analysis (GO), we identified several classes of genes that change simultaneously between the first and second trimesters (Fig. 1C–F; Tables S2-5). Notably, genes associated with protein processing in the endoplasmic reticulum (ER) were amongst the most differentially expressed, as were genes regulating cellular metabolism, hormone secretion, transport and the extracellular matrix (Fig. 1C–F). Changes for selected transcripts were validated at the protein level, using either western blotting or immunohistochemistry. To ascribe the DEGs to individual cell types the RNA-Seq results were compared with a published scRNA-Seq dataset (Liu et al., 2018) that included first-trimester (8 weeks) and late-second-trimester (24 weeks) samples. The scRNA-Seq identified several cell types in 8-week placentas, including cytotrophoblast (CTB), syncytiotrophoblast (STB), extravillous trophoblast (EVT) and stromal cells (STR), and EVT in 24-week placentas.

### Metabolism

Analysis of GO and Kegg pathway enrichment showed that genes associated with protein processing in the ER to be amongst the most differentially expressed between the two time points. The Kegg pathway ‘protein processing in ER’ was enriched in the first trimester (*P*adj.=1.63×10^−3^; Fig. S2), as were the GO terms ‘protein folding in ER’ (*P*adj.=6.31×10^−3^) and ‘regulation of protein secretion’ (*P*adj.=3.70×10^−8^; Fig. 1C–F). These, and other related GO terms suggest that ER functional activity is greater during the first than the second trimester, despite the relatively low oxygen concentration prevailing.

**Fig. 2.**
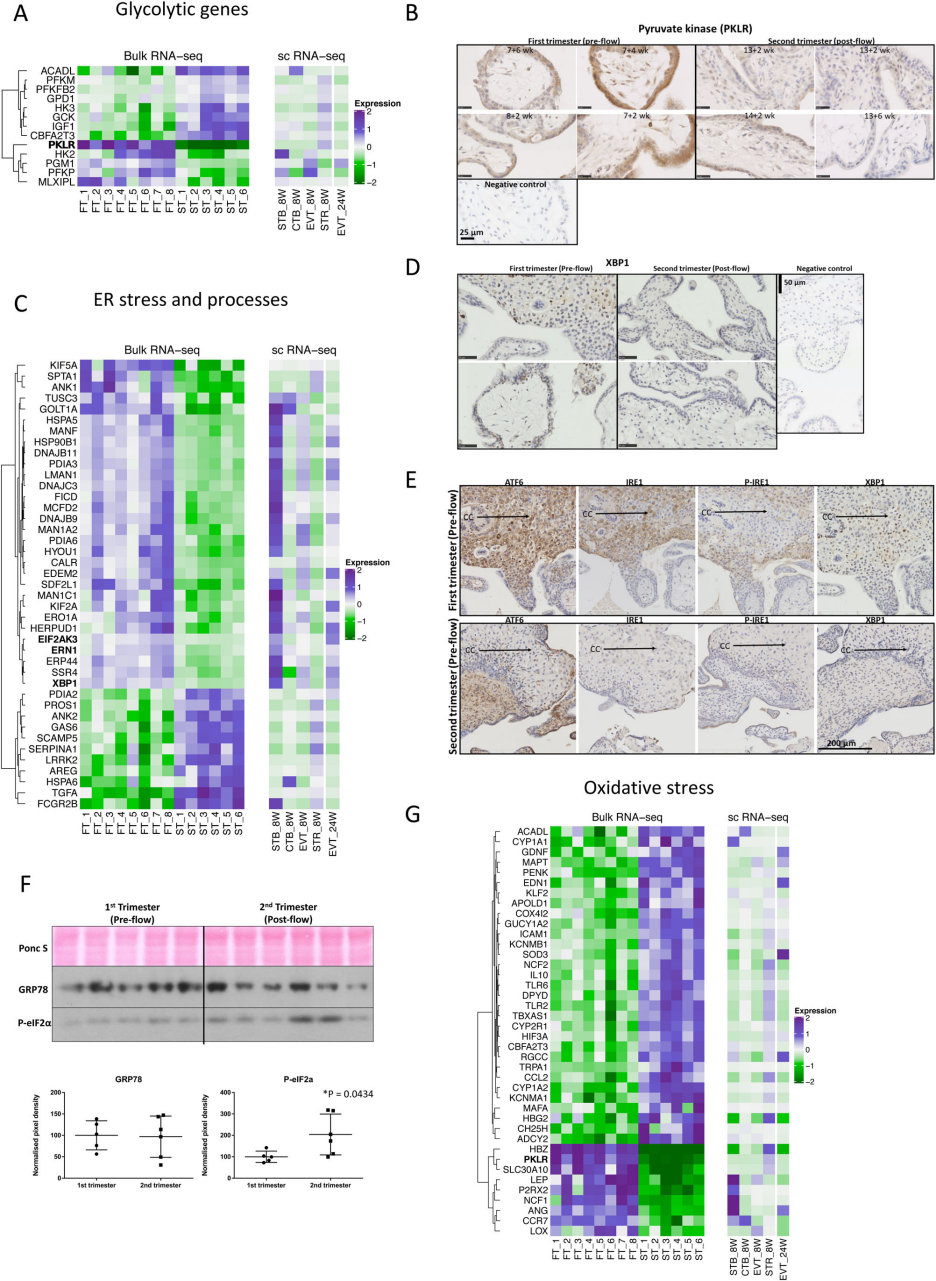
**DEGs associated with ER processing, oxidative stress and glycolytic processes.** (A) Heatmap of DEGs involved in the glycolytic pathway. Top DEGs in the first versus second trimester samples were compared to a previously published scRNA-Seq dataset (see Materials and Methods). (B) Immunostaining of first- and second-trimester sections with anti-pyruvate kinase antibody. (C) Heatmap of top DEGs in the first versus second trimester samples involved in ER stress and processes. Top DEGs were compared to a previously published scRNA-Seq dataset (see Materials and Methods). (D–E) Immunolocalisation of XBP1, ATF6, P-IRE1 and IRE1 in first- and second-trimester placental sections, stained with anti-XBP1, -ATF6, -IRE1 or P-IRE1 antibodies. Arrows denote cell columns (CC). (F) Western blots depicting GRP78 and phospho-eIF2α (E) or catalase (CAT) and glutathione peroxidate (GPX) (F) in first- and second-trimester placental lysates. Ponceau S (Ponc S) staining was used to normalise protein loading. Data are expressed as mean±s.d. Comparisons were made using a two-tailed Student's *t*-test. Differences were considered significant at *P*≤0.05. (G) Heatmap of top DEGs related to oxidative stress. Scale bars are 25 µm in B and D and 200 µm in E.

The transcript profile observed provides further evidence that placental tissues are not energetically compromised during the first trimester. Glycolysis is the primary route to energy generation, supported by the polyol pathways that preserve carbon skeletons for synthesis of purines and other molecules required for rapid cell proliferation (Burton et al., 2017). Consistent with this metabolic profile is the finding that *PKLR* and *HK2*, which encode the principal regulators of glycolysis, pyruvate kinase and hexokinase, are among the most differentially expressed genes (Fig. 2A–B). Pyruvate kinase is a key enzyme of the glycolytic pathway that catalyses the production of pyruvate and ATP from phosphoenolpyruvate. The synthesis of pyruvate kinase is controlled by two structural genes, the L-type gene (coding for the L and R isozymes) and the M-type gene (coding for the M1 and M2 isozymes), and four different mRNAs control the synthesis of pyruvate kinase isozymes. We detected a 5.3-fold change decrease in PKLR expression in the second trimester (Fig. 2A). The enzyme primarily localized to the syncytiotrophoblast of the first trimester villi (Fig. 2B). These findings are in agreement with those of Diamant et al. (1975) who reported elevated activity of pyruvate kinase in early gestation, indicative of high glycolytic potential, which tended to decrease with increasing gestational age.

Whilst present at low levels in most normal adult cells, *HK2* is highly expressed in embryonic tissues and cancer cells (Patra and Hay, 2013). Germ line deletion of *Hk2* in the mouse causes embryonic lethality (Heikkinen et al., 1999). We found *HK2* expression to be significantly higher in the first trimester, with a switch to the *HK3* isoform during the second trimester. HK2 follows Michaelis–Menten kinetics. It has low Km values for glucose, allowing for HK2 to be sensitive to accumulation of glucose-6 phosphate. In contrast, HK3 is inhibited by excess glucose and its substrate binding is attenuated by intracellular ATP (Cardenas et al., 1998).

Our data thus support the notion that glycolysis is replaced by oxidative mitochondrial respiration as the primary method of energy generation after the first trimester. Indeed, this conclusion is underpinned by the upregulation of the transcriptional co-repressor, *CBFA2T3,* at 13–14 weeks, which contributes to inhibition of glycolysis and stimulation of mitochondrial respiration (Kumar et al., 2013). Conversely, β-oxidation of fatty acids appears suppressed in the first trimester. Transcripts encoding long-chain acyl-CoA dehydrogenase (*ACADL*) rise in the second trimester (Fig. 2A). These changes may serve to protect the placental tissues from excessive production of reactive oxygen species when oxygen availability is low (Burton et al., 2017; Huang et al., 2014).

The ER is responsible for synthesis and post-translational modification of secreted and membrane proteins, and for intracellular Ca^2+^ storage. Loss of homeostasis activates the unfolded protein response (UPR), which is mediated by three signalling transducers: IRE-1 (inositol-requiring transmembrane kinase-endoribonuclease 1), PERK (PKR-like endoplasmic reticulum kinase) and ATF6 (activating transcription factor 6). Activity of these transducers is inhibited in the physiological state by binding of the ER chaperone protein BiP/GRP78/HSPA5. Transcripts encoding the sensor *IRE1*, known as *ERN1* (Fig. 2C), and its downstream X-box binding protein 1 (*XBP1*) were significantly higher in the first trimester (Fig. 2C–E), with no quantitative change in *GRP78* RNA or protein expression (Fig. 2F). There was no quantitative change in *ATF6* RNA expression but a subtle change in protein cellular localisation, as ATF6 immunostaining was observed almost exclusively in the cytotrophoblast cell columns during the first trimester, whilst it was localised less in the diminishing cell columns and more in the mesenchymal cells and syncytiotrophoblast in the second trimester (Fig. 2E). We also report a modest increase in *PERK* (*EIF2AK3)* (Fig. 2C; Fig. S2). In addition, transcripts encoding several heat shock proteins (*DNAJB9, DNAJB11, DNAJC3, HSPA5* and *HSP90B1*), those involved in protein post-translational modifications (*LMAN1, MAN1C1, MAN1A2, PDIA6, PDIA3* and *ERO1A*) and protein quality control (*ERP44, HERPUD1* and *EDEM2*), were also higher in the first trimester (Fig. 2C). These are likely to be homeostatic responses to a high synthetic activity.

Heat-shock proteins perform chaperone functions by stabilising new polypeptides, while PDIAs, ERO1A and MAN1s assist in disulphide bond formation and glycosylation to ensure correct folding of proteins. Activation of IRE-1 and its downstream target XBP1 are involved in the synthesis of lipoproteins essential for cell and organelle membranes. However, the actions of these pathways may be broader than just ER homeostasis. Activation of the IRE-1 pathway has been observed during development of the labyrinth zone of the murine placenta, with knockout of the gene leading to abnormal vascularisation (Iwawaki et al., 2009). We confirmed activation of the IRE-1/XBP1 pathway during the first trimester by immunostaining. XBP1 was strongly expressed by the villous and extravillous trophoblast of the cell columns, and XBP1 expression coincided with that of IRE-1 and P-IRE-1 (Fig. 2E).

As expected, there was upregulation of genes associated with oxidative stress and antioxidant defences during the second trimester, most notably *SOD3, HIF3A, COX4I2, CYP1A1, CYP1A2* and *NOS1AP* (Fig. 2G), in agreement with our previous findings (Jauniaux et al., 2000). Several GO terms associated with metabolism of oxygen were also significantly different between the gestational ages, including ‘reactive oxygen species metabolic process’ and ‘reactive oxygen species biosynthetic process’ (see Tables S3–S5). Consistent with these terms, we observed increased phosphorylation of eIF2α in the second trimester, indicative of activation of the UPR (Fig. 2F).

### Hormonal activity

**Fig. 3.**
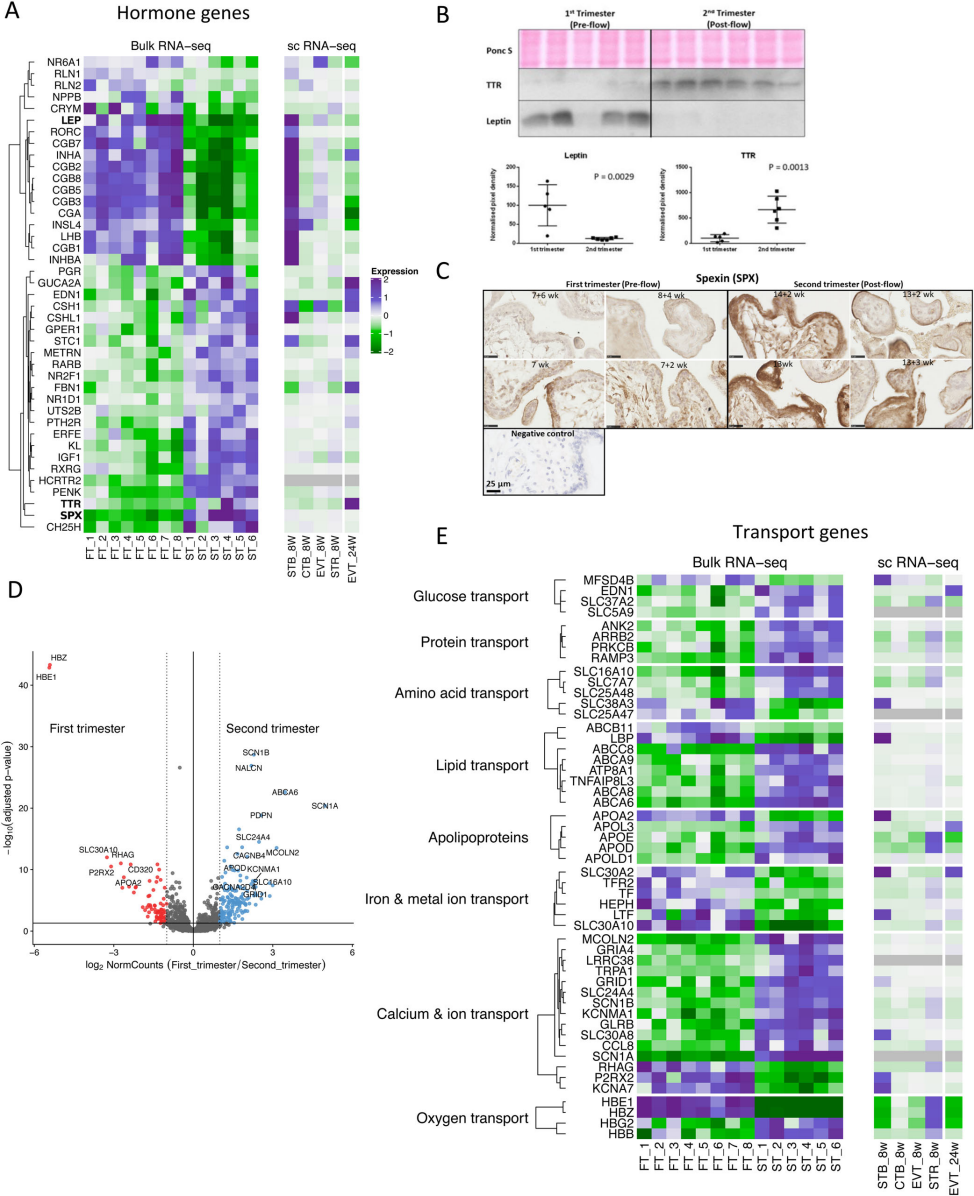
**Overview of hormonal and transport-related placental genes differentially expressed in the first and second trimesters.** (A) Heatmap showing top DEGs related to hormones and compared to the scRNA-Seq data. (B) Western blots depicting transthyretin (TTR) and leptin levels in first and second trimester placental lysates. Ponceau S (Ponc S) staining was used to normalise protein loading. Data are expressed as mean±s.d.. Comparisons were made using a two-tailed Student's *t*-test. Differences were considered significant at *P*≤0.05. (C) Immunostaining of first and second trimester sections with anti-spexin antibody. (D) Volcano plot of transport-related genes, with DEGs coloured red higher in the first trimester, and those in blue higher in the second trimester. (E) Heatmap of top transport-related genes, split by type of transport. Scale bars are 25 µm.

Transcripts encoding peptide hormones showed considerable differential expression (Fig. 3A,B). Transcripts higher at 7–8 weeks included subunits of hCG; *CGA* showed a 3.79-fold change while *CGB1*, *CGB2*, *CGB3*, *CGB5*, *CGB7* and *CGB8* showed fold changes of 3.68, 6.69, 3.84, 3.87, 4.94 and 3.59, respectively. These results confirmed previous findings that all six hCG genes are transcribed (Bo and Boime, 1992), and are consistent with secretion of hCG peaking at around 10 weeks of gestation. Secretion is likely to be stimulated by the epidermal growth factor receptor (EGFR) pathway (Wang et al., 2018). EGF is produced by the endometrial glands (histotroph pathway), and this signalling loop may be part of the trophoblast–endometrial dialogue stimulating early placental development (Burton et al., 2020). Also higher in the first trimester were transcripts encoding leptin (*LEP*) (fold change 3.16, Fig. 3A,B), relaxin (*RLN1*) (fold change 2.82) and insulin-like-4 (*INSL4*) (fold change 3.15). Comparisons with the scRNA-Seq data indicated the transcripts were enriched in the syncytiotrophoblast (Fig. 3A), the known site of production of hCG (Beck et al., 1986).

The greatest change in transcripts upregulated in the second trimester was for spexin (*SPX*) (fold change 11.08) (Fig. 3A,C). Immunostaining showed the hormone to be localised to the syncytiotrophoblast (Fig. 3C). The hypocretin receptor 2 (*HCRTR2*), also known as orexin receptor 2, is another pathway regulating appetite and lipid metabolism (Burdakov and Alexopoulos, 2005), and we found that its mRNA increased 8.55-fold between the first and second trimesters (Fig. 3A). In contrast, the transcript and protein levels of the hunger and satiety-maintaining hormone leptin were significantly higher in the first trimester placenta (Fig. 3A,B). Leptin concentrations are elevated during pregnancy due to an accumulation of maternal body fat and placental production. However, in contrast to leptin's effect on satiety, pregnancy requires increased food intake to meet the nutrient demands of the fetus and to lay down fat stores that are mobilised during late gestation and lactation. This apparent paradox is resolved by the development of central leptin resistance, which occurs in the second trimester of pregnancy (Grattan et al., 2007). Leptin mRNA and protein were found to colocalise to the syncytiotrophoblast and fetal endothelial cells in human placentas, suggesting that the placenta is a source of both fetal and maternal leptin (Lea et al., 2000).

Kegg pathway analysis showed that transcripts associated with ‘autoimmune thyroid disease’ and ‘parathyroid hormone synthesis, secretion and action’ were differentially expressed across the transition (Fig. 1C; Table S2). Thyroid hormones are important for early fetal development, in particular for the central nervous system, and must be transported across the placenta (Chan et al., 2009). Three major binding proteins, T4 binding globulin, transthyretin and albumin have been identified in the mature placenta (McKinnon et al., 2005). Here, we show for the first time that transcripts of *CRYM*, which encodes crystalline mu, a T3 binding protein, are present in the placenta and highest during the first trimester (2.97-fold change). By contrast, *TTR* encoding transthyretin is more highly expressed in the second trimester (2.9-fold change) (Fig. 3A,B). These findings suggest novel regulatory pathways for the transfer of thyroid hormones across the placenta, which change as pregnancy progresses.

### Transport

Transcripts encoding proteins mediating oxygen, lipid, protein, glucose and ion transport changed significantly (Fig. 3D,E). Transcripts encoding the hemoglobin subunits epsilon 1 and zeta (*HBE1* and *HBZ*) were within the top three differentially expressed genes, and were 43.16- and 42.47-fold lower in the second-trimester samples (Fig. 3D,E). HBE1 and HBZ make up embryonic hemoglobin that predominates during the first trimester. Conversely, the mRNA for hemoglobin G2 and hemoglobin beta (*HBG2* and *HBB*) were 2.98- and 3.49-fold higher in the later samples. HBB is a component of adult haemoglobin, while HBG2 is a component of fetal hemoglobin that is present at birth. These transcripts are likely to arise from the hemangioblastic clusters within the villous stromal core (Aplin et al., 2015), but both embryonic and fetal hemoglobin transcripts have been identified within purified CTB and EVT cells (Apps et al., 2011). Whether the changes in expression are determined by the rise in oxygen concentration, possibly protecting against oxidative stress (Nishi et al., 2008; Saha et al., 2014), or are ontogenetic is not known.

The pattern of expression of lipid transporters and apolipoproteins was profoundly different between the two trimesters. Transcripts highly abundant during the first trimester included *APOA2* and *ABCB11,* whereas those upregulated in the second trimester included *ABCA6, ABCC8* and *APOD* (Fig. 3E, Fig. S3). ApoD protects against ischemia-reperfusion injury in myocardial infarcts and has potent antioxidant activity, which may buffer the placenta once the maternal blood flow is established (Tsukamoto et al., 2013). Cholesterol is essential in early embryonic metabolism, cell signalling and elaboration of cell and organelle membranes, and must be transported across the placenta into the chorionic cavity. From there it is likely transported into the embryonic circulation via the secondary yolk sac, which expresses mRNAs encoding multiple apolipoproteins, the cholesterol efflux transporter ABCA1, and lipoprotein receptors, including megalin and cubilin (Cindrova-Davies et al., 2017). At the end of the first trimester, the chorionic cavity is obliterated by the enlarging amniotic cavity and the secondary yolk sac degenerates. Contemporaneously, onset of the maternal and fetal placental circulations permits transport of cholesterol and lipids across the villous membrane, and our data may reflect this change in the lipid transport pathway.

Transcripts encoding transporters of metal ions important for antioxidant defences were also higher in the first compared to the early second trimester (e.g. *SLC30A10, SLC30A2*, which had 9.56- and 3.14-fold changes, respectively). This differential might reflect higher transport of manganese and zinc ions that are essential cofactors for the superoxide dismutase enzymes during early pregnancy (Fig. 3E) in preparation for the rapid rise in pO_2_. Genes involved in the transport of iron also changed, in particular *LTF* that encodes lactotransferrin and *HEPH* that encodes hephaestin (3.68- and 4.07-fold changes, respectively).

Transcripts encoding ion channels, for example *SCN1A* (31.37-fold change), *MCOLN2* (8.83-fold change), *TRPA1* (7.38-fold change) and *SCN7A* (3.42-fold change) (Fig. 3E) were among those most significantly increased in the early second trimester. This may reflect a switch in the way amino acids are transported across the placenta. In the first trimester, they are transported by uptake and subsequent breakdown of proteins in maternal histotroph (Burton et al., 2002), whereas later in pregnancy there is active uptake of individual amino acids from the maternal circulation through accumulative and exchange transporters (Lewis et al., 2013). Activity of the latter needs to be balanced by other ionic fluxes.

### Cell proliferation, differentiation and WNT signalling

**Fig. 4.**
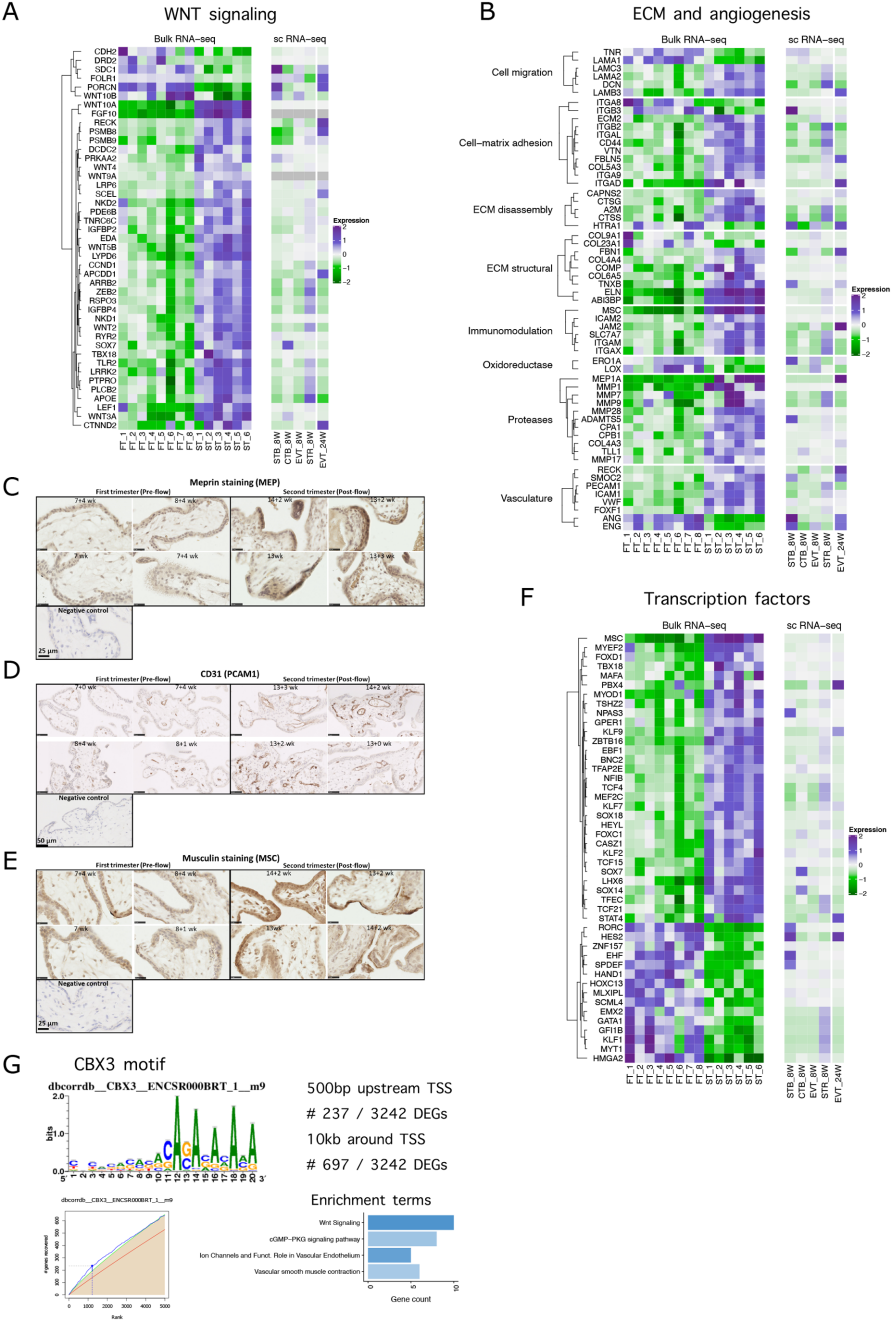
**Overview of DEGs related to transcription factors, WNT signalling and extracellular matrix related genes, differentially expressed between the first and second trimester.** (A) Heatmap of DEGs associated with WNT signalling. (B) Heatmap of extracellular matrix and angiogenesis-related DEGs. (C–E) Immunolocalisation of meprin (D), CD31 (E) and musculin (MSC) (F) in first- and second-trimester placental sections (*n*=4). Scale bars are 25 µm in C and E and 50 µm in D. (F) Heatmap of differentially expressed transcription factors. (G) CBX3 motif is enriched in proportion of DEGs: sequence motif for CBX3, incidence matrix of highly ranked DEGs with CBX3 motif, and their pathway enrichment analysis.

Recent evidence suggests that WNT signalling may be implicated in the regulation of placental development and human trophoblast differentiation (Haider et al., 2017; Nayeem et al., 2016). Our data show that *RSPO4*, *WNT10B* and several other genes mediating the canonical WNT signalling, including *PORCN* and *SDC1*, were higher in the first trimester than in the early second trimester (Fig. 4A). By contrast, *WNT3A, WNT10A, WNT2, LRRK2, RYR2, LRP6, CCND1* and *RSPO3* transcripts were significantly upregulated in the second trimester (Fig. 4A). Canonical WNT signalling has been shown to be critical for invasive trophoblast differentiation (Pollheimer et al., 2006). In addition, several genes that regulate the non-canonical WNT pathway were upregulated in the second trimester, including *WNT5B* and *LEF1*. Negative regulators of the WNT signalling, *NKD2* and *DKK3*, were also upregulated in the second trimester, suggesting that paracrine mechanisms play a role in regulating trophoblast invasion during the second trimester. The majority of the WNT-signalling transcripts upregulated in the second trimester were localised to EVT, whilst transcripts that were higher in the first trimester were localised to both EVT and the syncytiotrophoblast (Fig. 4A).

### Extracellular matrix and angiogenesis

Significant differences were found in transcripts regulating extracellular matrix (ECM) remodelling (Fig. 4B). In particular, laminin *LAMA1* was highly expressed in the first trimester, whereas *LAMB3, LAMA2, LAMC3* were upregulated in the second trimester. Laminins have an important role during implantation (Haouzi et al., 2011), maintenance of trophoblast stemness (Kiyozumi et al., 2020) and EVT migration (Zhang et al., 2018b).

Shortly after implantation, EVT migrate from the placenta into the endometrium where they are involved in the remodelling of the maternal spiral arteries that ultimately supply the placenta. The invasive properties of these cells are widely attributed to the matrix metalloproteinases 2 and 9 (Isaka et al., 2003; Staun-Ram et al., 2004). We found the mRNAs for *MMP9* (3.14-fold), *MMP1* (7.99-fold) and *MMP28* (3.77-fold) to be upregulated in the second trimester. *MEP1A* encodes for meprin, a member of the astacin family of metalloproteinases that was 2.46-fold higher in the second trimester. Meprins can be secreted, and thus may assist in matrix digestion, or be membrane bound, where they may be involved in extracellular cleavage of proteins (Sterchi et al., 2008). Meprins are expressed abundantly by epithelial cells of the intestine, kidney and skin, and we show for the first time that are located in the villous syncytiotrophoblast (Fig. 4C). We speculate they might be involved in remodelling the basement membrane during villous growth.

Vasculogenesis and angiogenesis are critical for successful placental exchange, and it has been suggested that the low-oxygen conditions during the first trimester stimulate these processes. However, we did not find classical hypoxia-regulated factors, such as *VEGF,* to be differentially expressed. This supports our previous findings showing that HIF protein is not stabilised during early pregnancy (Cindrova-Davies et al., 2015). By contrast, transcripts encoding other potent regulators of angiogenesis, angiogenin (*ANG*) and endoglin (*ENG*), were found to be higher during the first trimester (5.72- and 3.23-fold changes, respectively, Fig. 4B). Angiogenin mRNA and protein have been localised in the trophoblast and endothelial cells of the fetal placental vessels (Pavlov et al., 2014). In contrast, markers of vascularisation such as *PECAM1* (*CD31*)*, VWF, ICAM1* were significantly upregulated in the second trimester compared to the first trimester (Fig. 4B,D), indicating that the vascular components of the villi develop rapidly during the second half of pregnancy.

During pregnancy, the maternal immune system is modulated by signals from the placenta, with evidence of increased activation of innate cells in the systemic circulation. Regulatory CD4^+^CD25^+^Foxp3^+^ T cells (Tregs) expand during the second and third trimesters in the peripheral blood and in the decidua, believed to be induced by paternal antigens and contributing to the local control of fetus-specific maternal immune responses (Kahn and Baltimore, 2010; Rowe et al., 2012). The transcription factor musculin (MSC) is critical for the development of induced Treg cells by repression of the T helper type 2 transcriptional program (Wu et al., 2017). Transcripts for MSC were significantly upregulated in the second trimester (Fig. 4B,E). This is the first demonstration of the presence of this immune-regulator in the syncytiotrophoblast, although it may have a different function to its role in Tregs (Fig. 4E).

### Transcription factors

Rapid cell proliferation and differentiation occurs during the first trimester to establish the placenta. Transcripts encoding the transcriptional regulator high-mobility group AT-hook 2 protein (*HMGA2*) were 7.55-fold higher in the first trimester than the second (Fig. 4F). HMGA2 is known to play a role in proliferation and differentiation, and homozygous mutations in *Hmga2* result in the *pygmy* phenotype in mice (Zhou et al., 1995). We found that expression of *HAND1*, a transcription factor regulating differentiation of trophoblast subtypes in the mouse (Scott et al., 2000), was also higher in the first trimester (6.63-fold) (Fig. 4F), but its function during human placental development is unknown. Many of the transcription factors upregulated in the second trimester regulate mammalian development and differentiation processes. These include *KLF2*, *SOX14, SOX18, LHX6, MEF2C, SOX7, HEYL*, *TFAP2E, MYT1*, *BNC2* and *STAT4* (Fig. 4F).

To investigate potential regulatory networks of the placenta, we performed a motif rankings analysis. DEGs were scanned for the motifs, and DNA motifs significantly over-represented in a gene-set were identified. The CBX3 motif was the only common transcription factor motif that was significantly overrepresented in both searches (235 DEGs with CBX3 motif at 500 bp upstream of TSS and 691 genes with this motif in 10kb-centered around TSS) (Fig. 4G). We have compared our DEGs enriched with CBX3 motif with the targetome of CBX3 transcription factor, based on ENCODE Transcription Factor Target Database (Consortium, 2011; Rouillard et al., 2016). We found that 68 out of 237 DEGs with CBX3 motif within 500bp-TSS were already identified as CBX3 targets in that database. Some of the unidentified CBX3-enriched DEGs may be unique for the placenta, as many TF binding patterns are tissue-type specific.

The *CBX3* gene was slightly downregulated in the second trimester (-1.28-fold change, *P*adj. 0.0029). CBX3 plays a role in transcriptional silencing in heterochromatin-like complexes, and may contribute to the association of the heterochromatin with the inner nuclear membrane through its interaction with lamin B receptor. In mice, Cbx3 inhibits vascular smooth muscle cell proliferation, migration, and neointima formation (Zhang et al., 2018a). DEGs with the CBX3 motif (500bp-TSS) were extracted and pathway analysis with enrichR was performed. The top enriched term for WikiPathways_2019 and Kegg_2019 was ‘Wnt Signaling’ (*P*adj. 0.00049 and 2.794813e-03, respectively) (Fig. 4G), while the term ‘ion channels and their functional role in vascular endothelium’ (*P*adj.=0.042; Fig. 4G) was enriched from BioCarta_2016. These findings indicate that CBX3 could be the transcription factor, which links at least some of the differential changes taking place in the two trimesters, such as the differential regulation of the WNT signalling pathway that mediates EVT migration and trophoblast function, endothelial function and ion channel transport.

In addition to the CBX3 motifs and transcription networks, the motif for BCL6 was significantly overrepresented within 10 kb around TSS of DEGs (lower confidence motifs). The pathway analysis for DEGs enriched in BCL6 motif has shown similar terms to these for CBX3, i.e. ‘ion channels and their functional role in vascular endothelium’ from BioCarta (*P*adj. 0.0026) and ‘vascular smooth muscle contraction’ (*P*adj. 0.00765) from Kegg (Fig. S4). The BCL6 expression is significantly lower in the second trimester (-2.175-fold change, *P*adj. 0.018) and it has been previously shown to be required for proliferation of villous cytotrophoblast cells (Muschol-Steinmetz et al., 2016), whilst BCL6 overexpression reduces trophoblast fusion and is increased in pre-eclamptic placentas (Jasmer et al., 2017).

### DNA Methylation

**Fig. 5.**
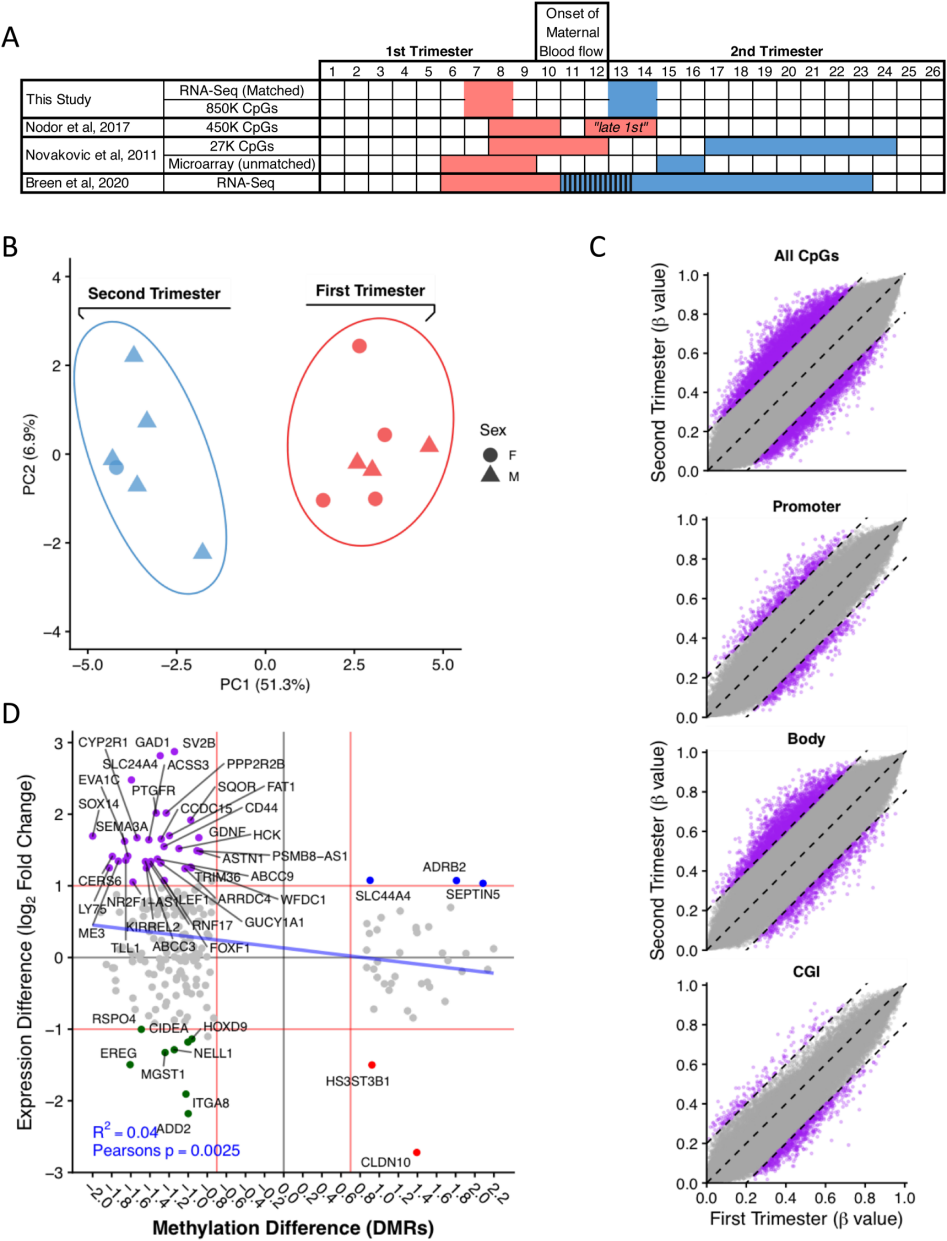
**DNA methylation changes in first versus second trimester.** (A) Comparison of gestational ages between this and related studies. (B) PCA shows clear separation between the first and second trimesters. (C) Global methylation levels are higher in the second trimester as indicated by a shift in points towards the top left. Methylation difference thresholds of 0.2 (20%) and 0.4 (40%) (dashed lines). At specific genomic features, the largest difference is observed at gene bodies with higher methylation during the second trimester, with increased numbers of probes with a methylation difference of over 0.2 (dashed lines). (D) Comparing RNA expression and DNA methylation at promoters and gene bodies shows correlation of reduced expression with increased methylation from first to second trimester for 36 genes (purple). Thresholds of log2 onefold change and a methylation difference of 0.75 (red line).

Placental DNA methylation increases over gestation, and *in-utero* exposures alter methylation and impact placental function and fetal health (Vlahos et al., 2019). Previous studies have compared first and second trimester methylation; however, these studies were not designed specifically to study the impact of onset of the maternal circulation inside the placenta. Thus, Novakovic et al. compared 8–12 week versus 17–24 weeks samples using a 27K methylation array focusing on gene promoters, but the first trimester samples overlap the first-second trimester transition (Novakovic et al., 2011). Nordor et al. compared 8–10 week to 12–14 week samples using a 450K methylation array, with the later time points overlapping the onset of maternal blood flow (Nordor et al., 2017). In both studies, gene expression data were not taken from the same samples, and standard bisulfite-treatment was used, which also includes a confounding 5-hydroxymethylcytosine (hmC) signal. In the present study, we extracted DNA from the same patient samples that we used for the RNA-Seq analysis. We have used an oxidative bisulfite treatment on samples assaying only 5-methylcytosine (mC). We also performed the methylation analysis using an EPIC array assaying over 850 K CpG sites with higher coverage to previous studies. Our study is therefore uniquely placed to elucidate the gene expression and methylation differences immediately before and after the full onset of maternal blood flow on the same samples (see Fig. 5A for a comparison of related datasets). Sex-specific effects were removed (see Materials and Methods).

Samples clustered separately by gestational age in a PCA using the most variably methylated CpG positions (*n*=500) (Fig. 5B). Mean CpG methylation across all positions assayed showed that there is a globally higher level of methylation during the second trimester (Fig. 5C). Previous studies have indicated the importance of promoter methylation (Novakovic et al., 2011; Nordor et al., 2017) and its positive correlation with gestational age. Our study compared the first and second trimester methylation levels across specific genomic features associated with gene regulation: promoters (2 kb upstream of TSS), gene bodies and CpG Islands. We found the largest difference within gene bodies (Fig. 5D). Differential methylation analysis between the first- and second-trimester samples revealed 329 DMRs, with 233 overlapping at least one gene promoter/body. We then correlated the differentially expressed genes from the RNA-Seq with differentially methylated CpGs in the same first- and second-trimester samples. After applying minimum thresholds of a twofold change (log2 fold change 1) for gene expression and a 20% methylation difference for DMRs, we found 49 DMRs overlapped DEGs. In the majority (36/49), increased gene expression was associated with a corresponding decrease in methylation at the promoter/gene body. Overall, a correlation of increased methylation with decreased expression was observed (R^2^ 0.04, Pearson's correlation coefficient *P*=0.0025) (Fig. 5D; Table S6). For example, increased *WNT2* expression correlated with reduced methylation in the second trimester (Fig. 5D), and may reflect the important role of canonical WNT signalling for the differentiation of invasive EVT (Pollheimer et al., 2006).

A GO term analysis suggests the differentially methylated genes are enriched (*P*adj.< 0.05) for molecular function terms related to in transcriptional regulation (Table S7). The GO analysis for biological processes are enriched for terms associated with cell fate commitment/specification, embryonic development/morphogenesis, regulation of metabolic processes and regulation of transcription (Table S8).

In the first trimester samples, reduced methylation correlated with higher expression of transcripts encoding the EGFR ligand, epiregulin (EREG), and *EREG* expression decreased after the full onset of maternal blood flow. EGF is abundantly secreted from the endometrial glands in early gestation when it stimulates cytotrophoblast proliferation and maintains their stemness (Burton, 2018; Maruo et al., 1992). Epiregulin promotes the cytotrophoblast-EVT transition through O-fucosylation on urokinase-type plasminogen catalysed by protein O-fucosyltransferase 1 (poFUT1) (Cui et al., 2019). This role seems critical for the pregnancy viability as both epiregulin and poFUT1 were reduced in placentas of patients suffering early pregnancy failure (Cui et al., 2019).

By contrast, expression of *CYP2R1* and *RBP7* was increased in the second trimester, with a corresponding decrease in methylation at this time. These genes regulate vitamin D and A metabolism; CYP2R1 converts vitamin D into its active form, whilst RBP7 affects vitamin A stability and metabolism. Adequate vitamin D function is essential for fetal skeletal development, tooth enamel formation and general fetal growth and development (Brooke et al., 1980), and vitamin A (retinoic acid) is essential for the development of heart, embryonal circulatory and central nervous systems and the regulation of heart asymmetry (Zile, 1998).

Decreased methylation levels were also correlated with the upregulation of genes activated in response to oxidative stress in the second trimester (*FAS, WFDC1, AOX1, CH25H*). Transcripts encoding several transporters mediating the uptake of sodium/potassium (*SLC24A4*), choline and thiamine pyrophosphate (B1 homeostasis; *SLC44A4*), organic anions and bile acids (*ABCC3*), and drugs (*ABCC9*) were increased in the second trimester, whereas magnesium and zinc transporter *SLC39A8* were increased in the first trimester. Methylation levels were differentially regulated in all these genes.

**Fig. 6.**
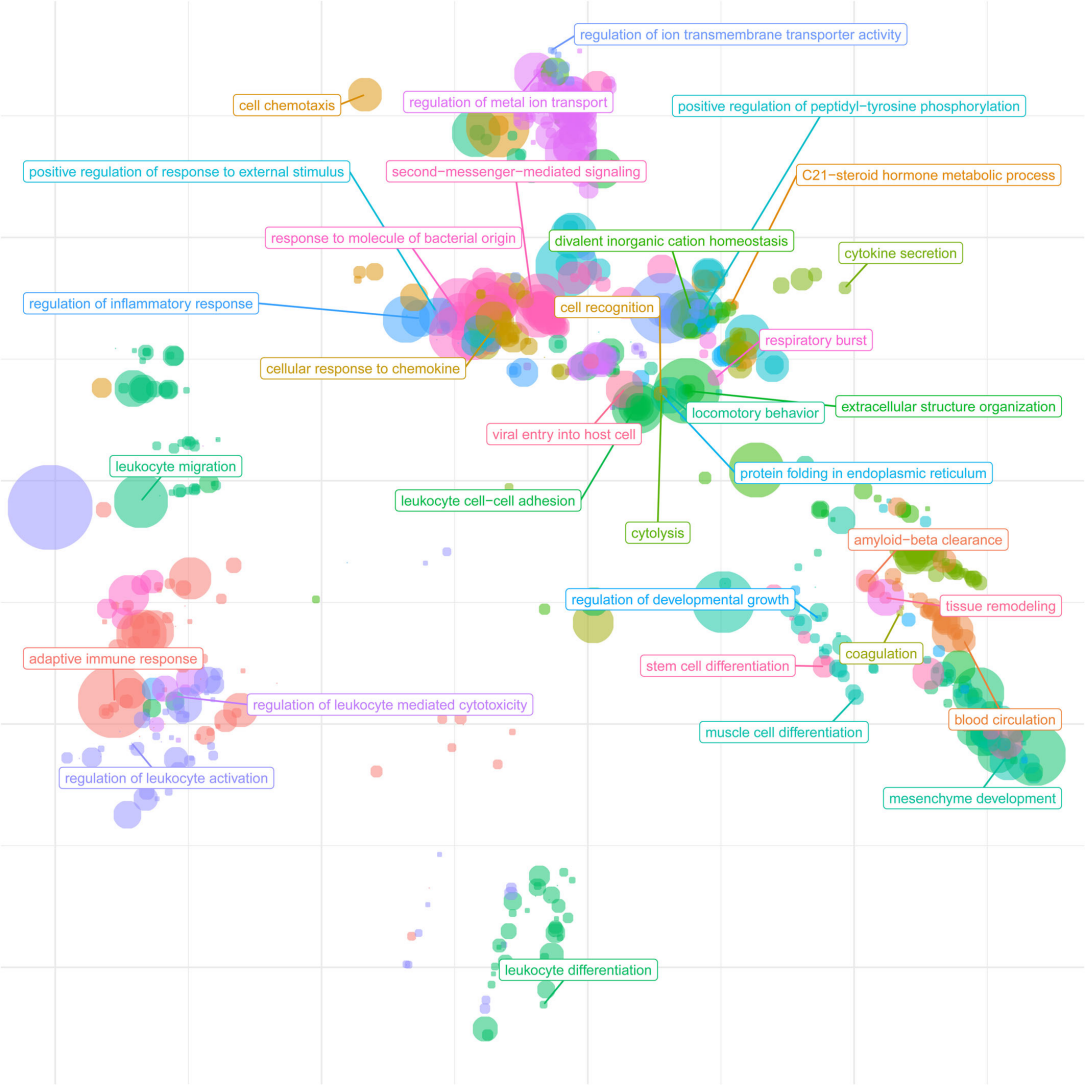
**Cluster analysis of** **biological process GO terms.** To summarise the significant GO terms related to biological processes of the differentially expressed genes between the first- and second-trimester groups, we performed a semantic similarity. Clusters of terms signify their biological relatedness. Main clusters emerge for transmembrane transporters, cell differentiation and development, and leukocyte and adaptive immune response.

The inverse correlation of DNA methylation with expression suggests an association for a subset of genes in response to the onset of blood flow. GO pathways in this subset of genes include signal transduction, anatomical structure development, cellular protein modification process, cell differentiation, small molecule metabolic process, response to stress, transport and immune system process (Table S8). We further extended the GO analysis to cluster related significant GO terms by their semantic similarity (Fig. 6) for a global view of biological processes changing between the first and second trimesters. Several clusters emerged related to transmembrane transporters, cell differentiation and development, and leukocyte and adaptive immune response, highlighting the key transcriptomic changes during the onset of maternal blood flow.

Interestingly, there was an overlap between the CBX3 targets and the differentially methylated regions of 22 genes, including WNT2, EREG, ABCC3, TLL1 and LY75 (Fig. S5). These findings elude to the potential role of CBX3 in transcriptional silencing of some of its targets.

## DISCUSSION

We sought to examine the effects of changes in the intrauterine environment that occur across the first–second trimester transition on the placenta at the levels of the transcriptome and methylome. The major changes centre around the onset of maternal arterial circulation, with the switch from histotrophic to hemotrophic nutrition. They include a threefold rise in oxygen concentration, together with increases in shear stress and other biomechanical stimuli at the villous surface. These constitute an environmental stress to which the placenta must successfully adapt (Jauniaux et al., 2000).

Our data reveal that expression of transcripts involved in the synthesis and ER processing of peptide hormones, including hCG, is high during the first trimester. These findings may initially appear paradoxical since protein synthesis is energy demanding; however, despite the initial low-oxygen environment there are no significant differences in placental concentrations of the main energy metabolites (ATP/ADP, NAD+, glucose and lactate) across gestation (Cindrova-Davies et al., 2015). This constancy is due to high activity of glycolytic pathways during the first trimester, as shown by the transcriptome profile here and supported by a plentiful supply of glycogen in the histotroph from the endometrial glands (Burton et al., 2017). A high output of hCG by the syncytiotrophoblast soon after implantation is essential to maintain the corpus luteum and prevent onset of the next menses. There is also a need for rapid cell proliferation to anchor the developing placenta into the uterine wall.

The corpus luteum is the principal source of progesterone and oestrogen until the syncytiotrophoblast takes over (Aspillaga et al., 1983). We found that the transcripts for two enzymes catalysing the conversion of cholesterol to progesterone (via pregnenolone), *CYP11A1* (P450scc) or *CYP17A1*, did not change significantly between the first and second trimesters. By contrast, the gene encoding cholesterol 25-hydroxylase (*CH25H*), which converts cholesterol to 25-hydroxycholesterol (25OHC), was significantly upregulated in the second trimester (Figs 2G and 3A), and appears regulated through methylation (Fig. 5D). 25OHC and other oxysterols are substrates of P450scc, and 25OHC enhances the production of steroids by the ovary and testis (Risbridger et al., 1986; Toaff et al., 1982). Increased expression of *CH25H* may contribute to the rise in placental progesterone synthesis at the end of the first trimester, which is pivotal to maintain the pregnancy.

The transcript pattern encoding proteins mediating transport of oxygen, lipid, protein, glucose, and ions changed significantly between the first and early second trimesters, reflecting increased oxidative stress and the onset of hemotrophic exchange between the maternal and fetal circulations. There were also increases in transcripts encoding steroidogenic enzymes, consistent with the placenta taking over from the corpus luteum, and in transcripts driving differentiation of the invasive extravillous trophoblast cells that anchor the placenta and are involved in remodelling of the spiral arteries that ultimately supply the placenta.

We identified the hormone spexin for the first time in the syncytiotrophoblast, and found it to be upregulated in the second trimester. Spexin is involved in the regulation of body weight and metabolism, and inhibits the uptake of long-chain fatty acids by adipocytes and hepatocytes (Kolodziejski et al., 2018). Synthesis of spexin by the villous trophoblast suggests that this protein may play a role in regulating maternal lipid metabolism during pregnancy, possibly making more fatty acids available for transport to the fetus. A recent study measured circulating spexin (SPX) during the course of pregnancy in women with gestational diabetes mellitus (GDM) versus healthy controls (Al-Daghri et al., 2019). The study does not provide direct comparison with ours, as the starting measurements were taken at 10.3 (±4.9) weeks and thus spanned both the first and second trimesters. However, the authors reported a significant increase in circulating levels of spexin in patients who developed GDM, with values correlating positively with glucose levels. Hence, placental spexin may influence maternal glucose use and availability during pregnancy.

Our finding that transcripts encoding the hypocretin receptor 2 are also increased during the second trimester suggests the placenta may play an important role in modulating maternal appetite and energy intake during pregnancy. An inverse correlation between circulating spexin and leptin levels was reported in adolescents with obesity (Kumar et al., 2018). Leptin mRNA and protein have been found to colocalise to the syncytiotrophoblast and fetal endothelial cells in human placentas, suggesting that the placenta is a source of both fetal and maternal leptin (Lea et al., 2000). In addition, human fetal adipose tissue is capable of producing leptin at the beginning of lipogenesis and differentiation (Atanassova and Popova, 2000). Placental leptin is transported bidirectionally at both fetal and maternal interfaces (Hoggard et al., 2001; Wyrwoll et al., 2005). Leptin may play other roles, for its receptor is expressed on invading extravillous trophoblast cells, and addition of leptin to cytotrophoblast cells in culture increases the production of matrix metalloproteinases (Castellucci et al., 2000). Hence, the hormone may stimulate trophoblast invasion, particularly during the first trimester. In addition, leptin is involved in the development and maturation of a number of organs, including the heart, brain, kidneys and pancreas, in animal models (Briffa et al., 2015). Placental leptin may therefore also play a role in stimulating organogenesis.

The motifs of two transcription factors, CBX3 and BCL6, were significantly overrepresented, indicating involvement of these transcription factor networks in the differential regulation of trophoblast migration, proliferation and fusion following onset of maternal blood flow. Aberrant expression of both transcription factors has been reported in many types of cancer (Polo et al., 2007; Zhao et al., 2019). It is possible there is an interaction between the two transcription factor networks that regulates trophoblast migration, proliferation and fusion following the onset of maternal blood flow. Supporting this notion is the fact that CBX3 is one of the target genes of the repressor gene BCL6 (Polo et al., 2007). In addition, cyclin dependent kinase inhibitor 1A (CDKN1A) is a target of CBX3 (Zhao et al., 2019) and is downregulated in the second trimester. CBX3 is known to be involved in transcriptional silencing in heterochromatin-like complexes. There are several differentially regulated growth regulating factors with CBX3 motif, including SCML4 (protein coding gene, downregulated in the second trimester), PRDM6 and 8 (histone methyltransferases), ZBTB16 (transcription repressor) and PRKCB (all upregulated in the second trimester).

Overall, the large number of differentially expressed genes (3260) identified demonstrates the extensive transcriptome changes occurring during the transition from first to second trimester, spanning the onset of maternal blood-flow. As summarised in Fig. 6, the key GO-term biological processes cluster by biological similarity into clusters for transmembrane transporters, cell differentiation and development, and leukocyte and adaptive immune response reflecting the extensive changes the placental must undergo with the switch from histotrophic to hemotrophic nutrition.

Our study has a number of strengths that limit potential confounding factors. Firstly, the placental samples were obtained using a CVS technique and frozen immediately, avoiding the activation of the stress response and HIF pathways when first-trimester placental villi are collected using suction curettage and exposed to maternal blood (Cindrova-Davies et al., 2015). Secondly, gestational age was accurately assessed by ultrasonography prior to the CVS procedure. Thirdly, the samples were taken from narrow windows of gestation prior to and following onset of the maternal circulation. The precise timing of onset in an individual pregnancy is not known, but the intraplacental oxygen concentration appears stable before 10 weeks and after 12 weeks of gestation (Jauniaux et al., 2000). Onset is also a progressive phenomenon, starting in the peripheral region of the early placenta where it stimulates villous regression and moving centripetally (Jauniaux et al., 2003b). Tissue effects are likely to be heterogeneous during the transition period, but our samples were all obtained from a consistent site, the central region of the placenta identified under ultrasound guidance, limiting regional variability. Finally, the RNA-Seq and methylation analyses were performed on the same samples.

The rigorous collection protocol meant, however, that sample sizes were relatively small. Nonetheless, some of the most DEGs identified, such as those for the various hCG and hemoglobin loci, were consistent with previous reports. In addition, we confirmed many of the novel findings, such as for PKLR, spexin, musculin and the meprins, at the protein level with immunohistochemistry. Our sample groups were also biased towards males. Hence, we performed a sex-adjustment analysis at the outset. The differentially expressed genes identified in this study did not intersect with those identified by Gong et al. (2018b), indicating that sex-specific effects were unlikely to drive the first–second trimester gene-expression differences observed.

The transcriptome can only provide an indication of potential protein levels and function due to differential translation and post-translational modifications. Nonetheless, it is clear that the transition between the first and second trimesters is associated with major physiological and morphological changes in the placenta. Villous regression over the superficial pole of the gestational sac leads to formation of the membranes and the definitive placenta, and there is a loss of stemness and proliferation of the villous cytotrophoblast cells (Burton et al., 2020). Premature onset of blood flow occurs in many patients with first-trimester miscarriage, suggesting that a failure to adapt successfully to the environmental changes may trigger pregnancy loss or subsequent complications (Jauniaux et al., 2003a). Our findings indicate that DNA methylation changes are part of the placental response to these major environmental changes and may explain the altered DNA methylation profiles seen in placentas from pregnancies complicated by fetal growth restriction (Hillman et al., 2015). Whether these changes are purely ontological or responsive to the change in oxygenation, the loss of growth factor support from the histotroph, or to increases in biomechanical stimuli requires further research.

The unique nature of the first–second trimester transition during human pregnancy may explain the high incidence of complications of pregnancy in our species. In other mammals, histotrophic and hemotrophic exchanges occur in separate areas of the extra-embryonic membranes, such as the inverted yolk sac and the chorioallantoic placenta of the mouse, respectively. In these species, the placenta develops in a more consistent oxygen environment, and the switch in nutrient pathways within the same tissue does not occur. The situation in the human reflects our invasive interstitial form of implantation, which is restricted to the great apes and for which the evolutionary advantage is yet to be identified.

## MATERIALS AND METHODS

### Human tissue collection

First- and second-trimester tissue samples were collected with informed written patient consent and approval of the Joint UCL / UCLH Committees on the Ethics of Human Research (05/Q0505/82) from uncomplicated pregnancies at 7–8 (*n*=8) and 13–14 weeks of gestation (*n*=6). Gestational age was confirmed by ultrasound measurement of the crown–rump length (CRL) immediately before the procedure. All samples were collected from patients undergoing surgical pregnancy termination under general anaesthesia for psychosocial reasons. Villous samples were obtained under transabdominal ultrasound guidance from the central region of the placenta using a chorionic villus sampling (CVS) technique. All samples were snap-frozen immediately in liquid nitrogen and stored at -80°C until analysis.

### RNA extraction and RNA-Seq

RNA was extracted from human first-trimester placental villi using the RNeasy Plus Universal Mini Kit (catalogue number 73,404; Qiagen). Libraries were made using the Illumina TruSeq Stranded mRNA Library Kit according to the manufacturer's instructions. Libraries were quantified (kappa qPCR), and equimolar pools were sequenced (paired end 100 base reads, PE100) in several lanes of the Illumina NextSeq.

### Bioinformatics

#### RNA-Seq analysis

Paired-end sequencing was performed on Illumina NextSeq Direct High Output with read lengths of 100 bp. QC of sequencing was assessed using FastQC (v 0.11.5), fastq_screen (v0.9.3) and Picard Tools (v 2.9.0) and summarised with MultiQC (v1.8dev). Reads were trimmed to remove adapters and low quality bases with TrimGalore! (v0.6.4) and aligned to the human genome (GRCh38) with STAR aligner (v2.5.1b), with a mean of 90.4% reads uniquely mapping and mean of 56 M paired reads/sample. Gene quantification was determined with HTSeq-Counts (v0.6.1p1). Additional quality control was performed with a custom rRNA and mtRNA counts script (provided on GitHub). Counts extracted with htseq-counts were used to perform differential gene analysis in R (version 3.5.2) using package DESeq2 (v.1.22.2). Sex of the samples was assigned using sex-specific gene expression *Xist, Rps4y1, Ddx3y, Usp9y* and *Sry* and was included in the design formula (∼ sex+condition) as a blocking factor to account for variation in the data. Additionally, DEG list was compared with the list of genes expressed differentially in placenta depending on fetal sex (Gong et al., 2018a). Among our list of 3242 genes (with fold change>2), there were none overlapping with the list (101 genes). Read counts were normalised on estimated size factors. Principal component analysis (PCA) was performed on rlog-transformed count data for all genes. GO and Kegg pathway analysis were performed using clusterProfiler package (v.3.10.1) on DEGs with absolute log2 fold change >1 and adjusted *P* value <0.05 were used. Kegg pathway analysis was performed for upregulated and downregulated genes separately. The data matrix for scRNA-seq data were obtained from the Wang lab (16) (GEO accession number GSE89497). Regularised log transformation function (from DESeq2 package) was applied to counts for heatmaps. Heatmaps were generated with ‘ComplexHeatmap’ R package (v 1.20.0). Selection of differentially expressed genes on heatmaps was based on highest significance (lowest adjusted *P* value) and highest absolute log2 fold changes. To investigate potential regulatory networks of the placenta, RcisTarget R package was used (v.1.2.1). Motif rankings for tss-centered-10 kb and 500 bp upstream of TSS were used in the analysis (human motif collection version 9, ‘mc9nr’, with 24453 motifs). DEGs were scanned for the motifs and DNA motifs significantly over-represented in a gene-set were identified. DEGs with enriched motifs were analysed with enrichR r package (v.1.0) for pathway enrichment. Semantic similarity of GO terms was performed with the rrvigo R package (v1.2.0), using significant Biological Process GO terms.

#### Integration of RNA-Seq with single cell RNA-Seq (scRNA-Seq)

The GSE89497 scRNA-Seq dataset downloaded as a matrix processed as described in Liu et al. (2018). The matrix was normalized and scaled in Seurat (Stuart et al., 2019) for use in the heatmaps.

### DNA Methylation analysis

#### Infinium methylationEPIC array

In order to compare the sequencing and methylation changes in the placenta, we extracted the DNA from the same patient samples, as those used for RNA extraction. Genomic DNA was isolated by QIAamp DNA mini kit (Qiagen, catalogue number 51304) following the manufacturer's instructions. Buffer AL (200 μl) was added to the sample, mixed by pulse-vortexing for 15 s, before incubating at 70°C for 10 min. Absolute Ethanol (200 μl) was then added to the sample, and mixed by pulse-vortexing for 15 s before transferring to the QIAamp Mini spin column and centrifuged at 6000 ***g*** for 1 min. The Mini spin column was washed once with Buffer AW1 (500 μl) following by Buffer AW2 (500 μl) before centrifuging at full speed for 1 min. For elution of genomic DNA, DNase-free water (100 μl) was added and incubated for 1 min before centrifuging at 6000 ***g*** for 1 min. The step repeated one more time with another 100 μl DNase-free water. DNA concentration of the samples were quantified by NanoDrop and the DNA quality was checked by resolving in 0.8% agarose gel, in which there was a major band visualised at around 10 kbp without obvious smear below, indicating good quality DNA.

Genomic DNA oxidative bisulfite (oxBS) conversion was performed using the CEGX TrueMethyl kit (Cambridge Epigenetix/NuGEN, cat. no. CEGXTMS) and used for microarray-based DNA methylation analysis, performed at GenomeScan (GenomeScan B.V., Leiden, The Netherlands) on the HumanMethylation850 BeadChip (Illumina, Inc., San Diego, CA, USA). The EPIC array interrogates approximately 865,000 CpG sites representing about 99% of the RefSeq genes. The resulting iDAT files were imported and analysed using ChAMP (v2.9.10) (Aryee et al., 2014; Morris et al., 2014). Samples were processed filtering for a probe detection *P* value≤0.01, probes with a beadcount <3 in at least 5% of samples, no CpG and known SNPs (Zhou et al., 2017) at probe starts, probes aligning to multiple locations, and QC using the on array control probes. In total, 750150 probes on the array passed the filtering and QC steps. The BMIQ (Teschendorff et al., 2013) method was used to normalise the two probe types (I and II) present on the array. Beta methylation values from the EPIC array range from 0 (unmethylated) to 1 (methylated) and are equivalent of percentage methylation.

To account for sex specific differences we performed batch correction with champ.runCombat, using sex as the variable for correction. The PCA plot was generated for the top 500 most variably methylated CpGs. DMRs were calculated using the bumpHunter methods in ChAMP, and a methylation difference of 0.2 and adjusted *P* value of 0.05 between first and second trimesters was used for filtering DMRs. A threshold of 0.2 (20%) was used to select methylation differences above the cell-type variation within the placenta samples. A 20% threshold has been chosen in veriaous studies including in autoimmune disease (Lanata et al., 2018), CRISPR screens (Farris et al., 2020) and cancer (Dominguez et al., 2015). Probes on the X and Y chromosomes were excluded to minimise sex specific in differential methylation calculations. Pearson's correlation (R function cor.test) was used to calculate *P* values between DMR methylation and differential gene expression was calculated Pearson's. Bedtools (Quinlan and Hall, 2010) was used to determine DMRs overlapping gene bodies and promoters (bedtools closest -D b -d) -a DMRs.bed -b GRCh37.87.gtf.bed and 1.5Kb upstream of the TSS used to define promoters). GO analysis on for DMRs in gene bodies and promoters was performed with ‘goregions’ from missMethyl (Phipson et al., 2016). Multiple DMRs per gene were manually checked and in all cases the methylation change was in the same direction. Where a DMR overlapped two genes, both were included in the correlation with gene expression. A list of the top 100 DMRs associated with sex-specific methylation in human placentas are from Gong et al. (2018a). An intersection of sex-specific DMRs with the identified DMRs with associated expression changes above thresholds (log2 FC 1, methylation difference 0.2) show no common genes, suggesting the identified DMRs are not sex-specific.

### Data and code availability

#### RNA-Seq

Scripts used can be found on GitHub (https://github.com/CTR-BFX/2020_Prater_Cindrova). RNA-sequencing data is accessible through the EMBL-EBI ArrayExpress accession number: E-MTAB-9203. http://www.ebi.ac.uk/arrayexpress/experiments/E-MTAB-9203.

#### DNA Methylation (EPIC Array)

Code used to analyse the EPIC array samples is available on GitHub (https://github.com/CTR-BFX/First-Second-Trimester-Methylation). EPIC methylation array data have been deposited at EMBL-EBI ArrayExpress under accession number E-MTAB-9312 (https://www.ebi.ac.uk/arrayexpress/experiments/E-MTAB-9312).

### Immunohistochemistry

Immunohistochemistry was performed as previously described (Cindrova-Davies et al., 2007) using the following primary antibodies: anti-XBP1 (ab109621, Abcam), anti-phospho-IRE1 (ab48187, Abcam), anti-IRE1 (ab37073, Abcam), anti-ATF6 (ab37149, Abcam), anti-pyruvate kinase L/R (PB9499, Boster), anti-spexin (H-023-81, Phoenix Pharmaceuticals), anti-MEP1α (ab232892, Abcam), anti-CD31 (M0823, Dako), anti-musculin (ab64954, Abcam). Negative controls were performed by replacing primary antibodies with the blocking serum.

### Western blotting

Placental lysates were processed and run on western blots, as previously described (Cindrova-Davies et al., 2007), using the following antibodies: anti-TTR (PA5-80197, Thermo Fisher Scientific), anti-leptin (ab2125, Abcam), anti-ApoA1 (PA5-78798, Thermo Fisher Scientific), anti-eIF2α (#3398, Cell Signaling), anti-GRP78 (610978, Transduction Laboratories), anti-GPX1 (ab167989, Abcam).

## Supplementary Material

Supplementary information
