# Supplementary material for: RNA-Seq reveals changes in human placental metabolism, transport and endocrinology across the first–second trimester transition
Source: Biol Open. 2021 Jun 8;10(6):bio058222. doi: 10.1242/bio.058222 (PMC8214423; doi:10.1242/bio.058222)
Supplement: Supplementary information [file biolopen-10-058222-s1.pdf]

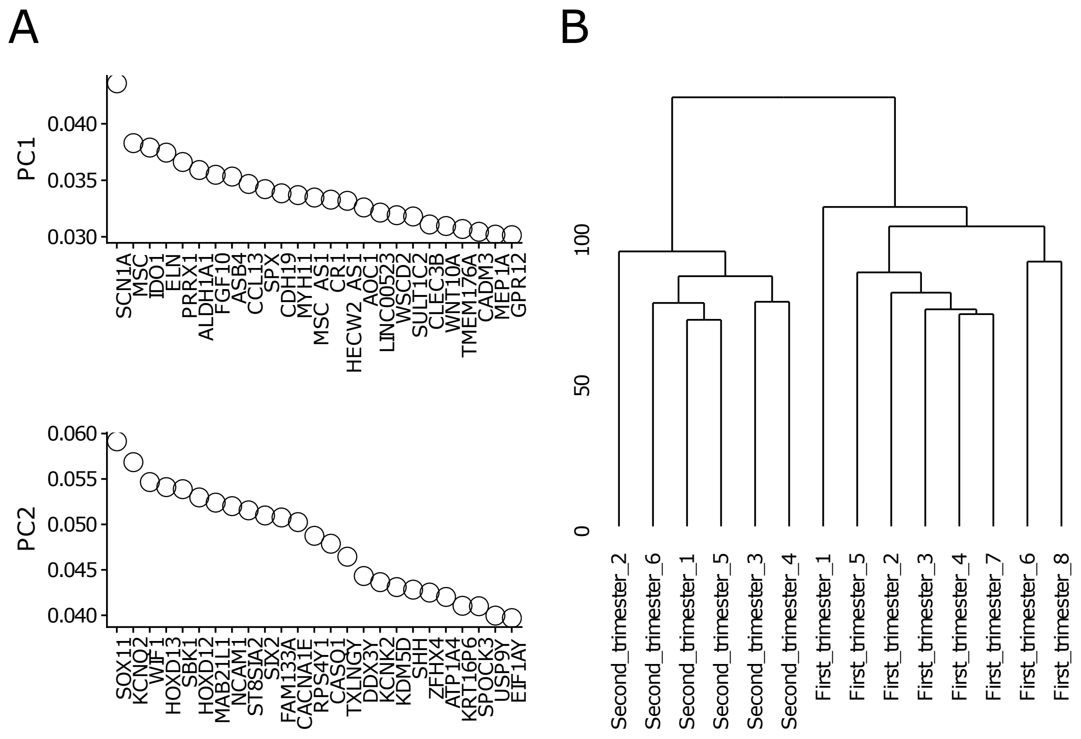

Figure S1. Principle Component Analysis Variance Explained.

A. PC1 and PC2 loadings showing a ranked list of genes to most contributing to the separation of groups on PCA plot. B. hierarchical clustering plot using showing clear separation of first and second trimester samples using rlog transformed counts.

Biology Open • Supplementary information

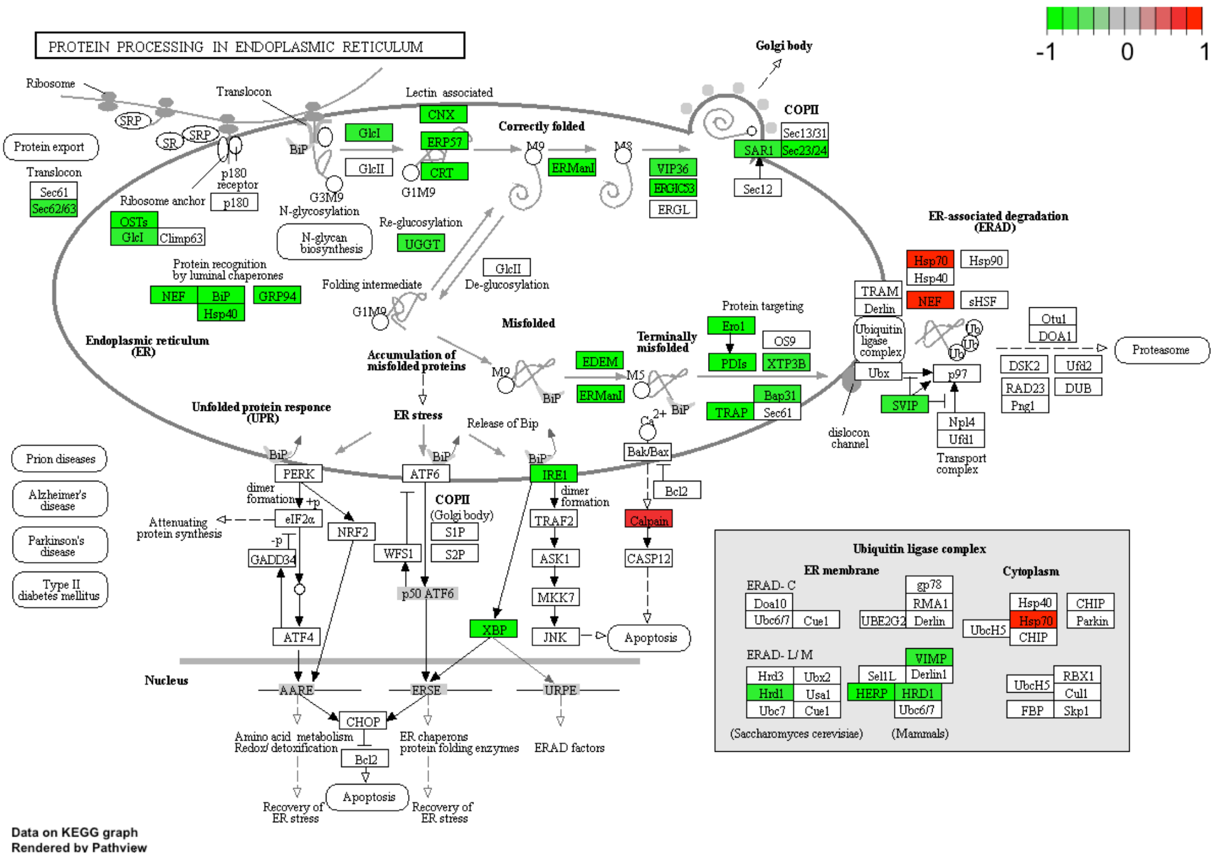

Figure S2. Protein Processing in the Endoplasmic Reticulum. Graphical representation of Kegg pathway term “Protein Processing in Endoplasmic Reticulum”, with genes expressed more in first trimester marked in green, and genes expressed more in second trimester in red.

Biology Open • Supplementary information

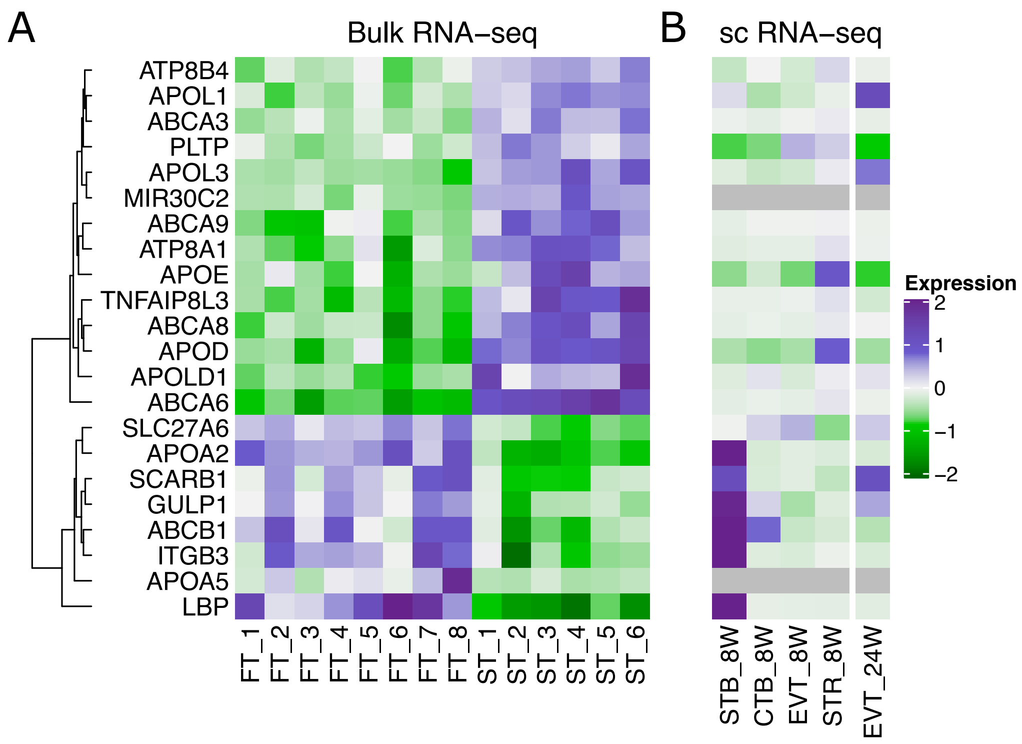

Figure S3. Lipid Transport Genes.

A. Heatmap of differentially expressed genes related to lipid transport (abs fold change > 1.5, FT= first trimester, ST= second trimester). **B.** A comparison to scRNA-Seq to identify which cell types express the genes (GEO GSE89497, STB= Syncytiotrophoblast, CTB= Cytotrophoblast, EVT=extravillous trophoblasts at 8 or 24 weeks).

Biology Open (2021): doi:10.1242/bio.058222 Supplementary information

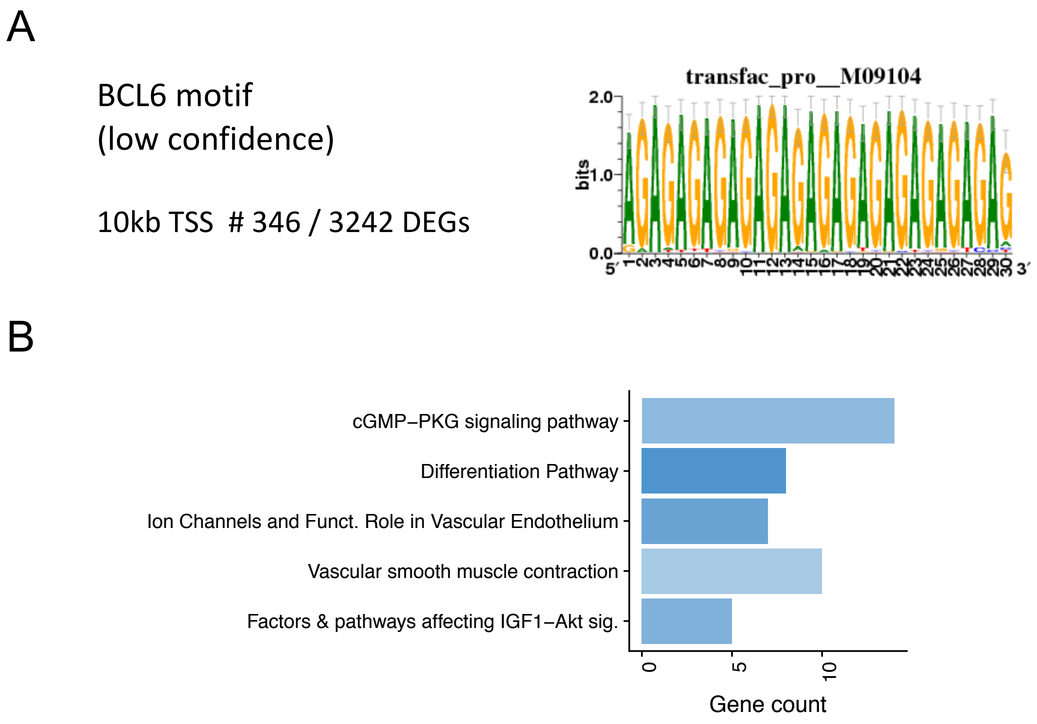

Figure S4. Transcription Factor Prediction.
A. B-cell lymphoma 6 protein (BCL6) motif is enriched in proportion of DEGs. **B.** In the panel are: sequence motif for BCL6 and pathway enrichment analysis for these genes.

Biology Open • Supplementary information

A

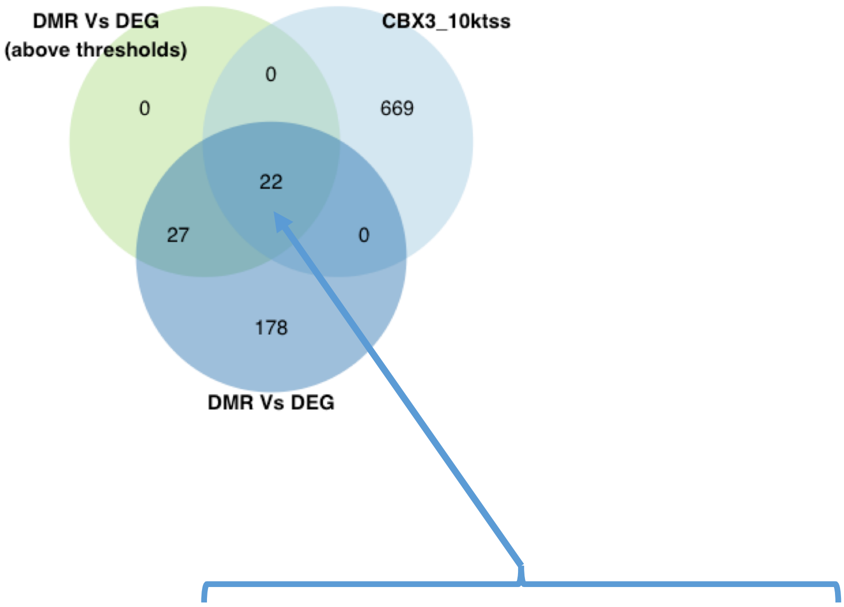

B

ensEML ID	gene	description
ENSG00000122420	PTGFR	prostaglandin F receptor
ENSG00000152092	ASTN1	astrotactin 1
ENSG00000077943	ITGA8	integrin subunit alpha 8
ENSG00000165973	NELL1	neural EGFL like 1
ENSG00000134873	CLDN10	claudin 10
ENSG00000140090	SLC24A4	solute carrier family 24 member 4
ENSG00000125430	HS3ST3B1	heparan sulfate-glucosamine 3-sulfotransferase 3B1
ENSG00000108846	ABCC3	ATP binding cassette subfamily C member 3
ENSG00000176194	CIDEA	cell death inducing DFFA like effector a
ENSG00000126259	KIRREL2	kirre like nephrin family adhesion molecule 2
ENSG00000054219	LY75	lymphocyte antigen 75
ENSG00000128683	GAD1	glutamate decarboxylase 1
ENSG00000124882	EREG	epiregulin
ENSG00000138795	LEF1	lymphoid enhancer binding factor 1
ENSG00000038295	TLL1	tolloid like 1
ENSG00000083857	FAT1	FAT atypical cadherin 1
ENSG00000168621	GDNF	glial cell derived neurotrophic factor
ENSG00000152503	TRIM36	tripartite motif containing 36
ENSG00000156475	PPP2R2B	protein phosphatase 2 regulatory subunit Bbeta
ENSG00000169252	ADRB2	adrenoceptor beta 2
ENSG00000075213	SEMA3A	semaphorin 3A
ENSG00000105989	WNT2	Wnt family member 2

Figure S5. CBX3 Transcription Factor Motif Enrichment in DEGs.
A. Venn diagram comparing overlap of differentially expressed genes (DEGs), differentially methylated regions (DMRs) between first and second trimester. Threshold of log2FC > 1.5 and adjusted p.value < 0.05. **B.** List of DEGs enriched containing the Chromobox protein homolog 3 (CBX3) motif.

Biology Open • Supplementary information

Tables S1-S8

Click here to download Tables S1-S8

Biology Open • Supplementary information
